# Differences in gait analysis and clinical outcome after TightRope® or screw fixation in acute syndesmosis rupture: study protocol for a prospective randomized pilot study

**DOI:** 10.1186/s13063-020-04550-5

**Published:** 2020-07-02

**Authors:** Julian Doll, Stefan Waizenegger, Thomas Bruckner, Gerhard Schmidmaier, Sebastian I. Wolf, Christian Fischer

**Affiliations:** 1 Clinic of Orthopedics, Trauma Surgery and Spinal Cord Injury, Heidelberg University Hospital, Schlierbacher Landstrasse 200a, 69118 Heidelberg, Germany; 2 Institute of Medical Biometry and Informatics, University of Heidelberg, D-69118 Heidelberg, Germany

**Keywords:** Syndesmosis rupture, TightRope®, Syndesmotic screw fixation, Gait analysis

## Abstract

**Background:**

Ankle sprains and fractures are most common injuries in orthopedic and trauma surgery. The concurrent occurrence of syndesmosis ruptures in these injuries represents a more complex problem, as they often remain undetected.

A proper and accurate treatment of injuries of the syndesmosis, both isolated and combined with fractures, is necessary to avoid long-term consequences (chronic instability, cartilage damage, and post-traumatic osteoarthritis). The most popular treatment option is a static screw fixation and the newly developed dynamic TightRope® (Arthrex, Naples, FL, USA).

The aim of this pilot study is to compare monitor ankle range of motion and maximum ankle power in gait as functional outcome parameters of instrumented gait analysis, as well as clinical and radiographic outcome for assessing the stabilization of acute syndesmosis rupture with either a static implant (a 3.5 mm metallic screw) or a dynamic device (TightRope®).

**Methods:**

This prospective, randomized, controlled, clinical trial will be carried out at the Center for Orthopedics, Trauma Surgery and Spinal Cord Injury of the University Hospital Heidelberg. Adult patients, who suffer from an acute syndesmosis rupture, both isolated and in combination with fractures of the lateral malleolus (Weber C and Maisonneuve fractures) and who are undergoing surgery at our trauma center will be included in our study. The patients will be randomized to the different treatment options (screw fixation or “TightRope®”). Subsequent to the surgical treatment, all patients will receive the same standardized follow-up procedures including a gait analysis and MRI of the ankle at 6 months follow-up.

The primary endpoint of the study is the successful healing of the syndesmosis and biomechanical investigation with gait analysis.

**Discussion:**

The results of the gait analysis from the current study will help to impartially and reliably evaluate the clinical and biomechanical outcome of both treatment options of acute syndesmosis ruptures. We hypothesize that the dynamic fixation provides an equivalent or better biomechanical, clinical, and radiographic outcome in comparison to the screw fixation.

**Trial registration:**

German Clinical Trials Register (DRKS) DRKS00013562. Registered on July, 12, 2017.

## Background

Ankle fractures are common injuries in the clinic and operative daily routine of a trauma surgeon [1]. Syndesmosis ruptures occur in 13% of these fractures and represent a more complex problem [2]. The syndesmosis ligament connects the tibia and the fibula above the ankle joint. It is, among other ligaments, responsible for its stabilization. Isolated injuries of the syndesmosis are extremely rare and often remain undetected in the clinical routine [3]. There are several clinical tests (Frick test, squeeze test, cross-leg test, fibula translation test) which confirm the suspicion of injury, but cannot secure it. Although certain syndesmotic injuries may be diagnosed radiographically, these injuries are often missed because of the inability of radiographs to detect them. Tibiofibular clear space greater than 6 mm (diastasis) in AP X-ray view is considered as a radiologic criterion for instability.

Today, magnetic resonance imaging (MRI) is the “gold standard” for the pretherapeutic evaluation of syndesmotic injury with a sensitivity of 100% and a specificity of 93% [4].

A proper treatment of injuries of the syndesmosis complex is challenging but necessary to avoid malreduction of the syndesmosis that alters tibiofibular joint kinematics and leads to chronic instability, cartilage damage, and early osteoarthritic changes of the ankle joint [5, 6]. Therefore, accuracy and maintenance of reduction of the syndesmosis are considered essential when treating ankle fractures with concomitant syndesmosis injury.

Even small joint gaps, axis deviations, or instabilities lead to considerable dysfunctions and thus increase the risk of post-traumatic arthrosis development [7].

The most popular treatment option of the unstable distal tibiofibular joint is a static 3.5-mm screw fixation (FA DePuy Synthes, USA) with one or multiple screws through three or four cortices. Disadvantages of the syndesmosis screws are partial weight-bearing for at least 6 weeks, neglect of the dynamic property of the syndesmosis, and an increased risk of chronic instability as well as the potential of late diastasis due to loosening, screw breakage, or screw removal. Furthermore, syndesmosis malreduction is reported to occur in up to 50% with syndesmotic screw fixation [8, 9]. Six to 8 weeks after the initial treatment, a mandatory implant removal is required to begin weight-bearing at our trauma center. In contrast, some authors prefer to retain the syndesmotic screw in place until she breaks. Whether or not the syndesmotic screw should be removed prior to weight-bearing is still discussed [10].

In contrast to screw fixation, the flexible, dynamic TightRope® (Arthrex, Naples, FL, USA) suture button device was developed for physiological stabilization of the distal tibia and fibula. The use of this dynamic suture button device has increased rapidly over the last years. Theoretically, this suture button device allows physiological motion of the syndesmosis without need for implant removal, which may reduce the risk of recurrent syndesmotic diastasis as described after syndesmosis screw removal [11, 12].

Biomechanical investigations have demonstrated that the strength of TightRope® device is comparable to a tricortical 3.5-mm syndesmotic screw. Several recent studies assessed syndesmosis stabilization with the suture button device and comparative studies reported equivalent or better functional results in comparison to the syndesmotic screw [3, 13–16].

Instrumented gait analysis is a well-established procedure at our trauma center, which allows objective quantification of gait deviations in the context of conservative and surgical treatment in the lower extremity. For assessment, the patient is equipped with skin-based markers and asked to walk repeatedly along a 5-10-m-long walkway at self-selected speed for monitoring joint motion via optical motion tracking. Floor mounted force plates allow for determining ground reaction forces when walking across them. This so-called “standard clinical instrumented 3D gait analysis” is a modern in vivo analysis, which allows for analyzing joint motion and joint kinetics of the major lower limb joints [17, 18].

To date, no study has yet compared both treatment options in terms of biomechanical outcome with kinetic, kinematic changes, and compensation mechanisms.

The majority of earlier studies of syndesmosis fixation, which demonstrated the equality of both treatment options, used only standardized questionnaires and plain radiographs to assess syndesmosis reduction and clinical outcome.

Therefore, the purpose of this prospective randomized controlled single-center study was to assess the functional outcome namely by ankle range of motion as well as maximum ankle power as obtained via gait analysis and to rate the clinical and radiologic outcome by monitoring the stabilization of acute syndesmosis rupture with either a static implant (a 3.5-mm metallic screw through three cortices) or a dynamic device (TightRope®).

In this study, we expect that dynamic fixation would provide an equivalent or better clinical and radiologic outcome as well as a similar or better function of the ankle, i.e., larger range of ankle motion within the gait cycle and a larger maximum plantar flexion power at pre-swing. It can be assumed that when the maximum ankle performance normalizes, there is sufficient healing.

This study focuses mainly on the treatment of syndesmosis rupture, but it also aims to gain insights into patients’ satisfaction in correlation to functional outcome and surgical technique. Additionally, the study targets to show how risk factors, different therapeutic modalities, and/or treatment strategies influence this outcome. In future, the risk of long-term consequences and complications should be minimized by analyzing the new and already existing data, the clinical-functional follow-up examination, and the subjective result.

The study protocol for this study is described in the present manuscript.

## Study design/methods

### Objectives and hypotheses

The objective of this prospective randomized controlled single-center study is the comparison of gait analysis, clinical and radiographic outcome after stabilization of an acute syndesmosis rupture with either a static implant (a 3.5-mm metallic screw through three cortices) or a dynamic device (TightRope®). Due to the pilot study design, further objective is to generate additional possible hypotheses.

The following hypotheses will be tested:

- H0: The biomechanical (regarding ankle range of motion and maximum plantar flexion power in the gait analysis), clinical (study questionnaire, SF-12, OMAS, VAS, FAOS), and radiological outcome is different in both treatment options.
- H1: The biomechanical (regarding ankle range of motion and maximum plantar flexion power in the gait analysis), clinical (study questionnaire, SF-12, OMAS, VAS, FAOS), and radiological outcome is equal in both treatment options.

Patients with acute syndesmosis rupture (isolated or combined), suitable according to the study protocol, will be randomized to the two different treatment options (screw fixation or TightRope®).

Syndesmosis reduction will be assessed using CT/3D imaging intraoperatively with ISO C 3D or CIOS Spin (Siemens Healthineers GmbH, Erlangen, Germany) as a part of our standard procedure.

Six months after the initial treatment, the patients will receive an additional gait analysis and a MRI in the follow-up examination at our outpatient clinic

### Study design, registration, and ethics

The study protocol was conducted according to the Declaration of Helsinki and approved by the local ethical committee of our hospital (S-454/2017). Furthermore, it was registered at the German Clinical Trials Register (DRKS00013562).

This study is a registered, clinical, prospective, randomized, controlled, single-center, two-arm, parallel group trial with a 1-year follow-up, carried out at a level 1 trauma center.

### Inclusion and exclusion criteria

Patients older than 18 years who suffer from acute syndesmosis rupture, both isolated and in combination with fractures of the lateral malleolus (Weber C and Maisonneuve fractures) and who are undergoing surgery within 8 days for a syndesmotic rupture at our Center for Orthopedics and Trauma Surgery will be included in this study after giving their informed consent.

Exclusion criteria are pregnancy, the disability for approval, congenital deformities of the lower extremities, missing informed consent, and refusal of participation. Patients with any known contraindications for MRI examination will be excluded as well.

### Study setting and population

Study patients pass a follow-up over 1 year with clinical and radiologic examinations 1 day before and after the initial operation as well as 6, 12, 26, and 52 weeks after treatment in our outpatient clinic. In this study, there is no need for additional clinical examinations for the patients, with the exception of an additional motion analysis and a MRI 6 months after initial treatment to evaluate the biomechanical outcome and the healing status of the syndesmosis ligament.

Surgery and examinations will be performed by an experienced orthopedic and trauma surgery consultant.

The patients will be randomized to the different treatment options (screw fixation or TightRope®).

The following questionnaires will be used for clinical and psychosocial evaluation:

- “Study questionnaire screw vs. TightRope”.
- “SF-12 – Health survey” [19].
- “Score of Olerud und Molander (OMAS)” [20].
- “Pain on a VAS”.
- “Foot and ankle outcome score (FAOS)” [21].

**Fig. 1:**
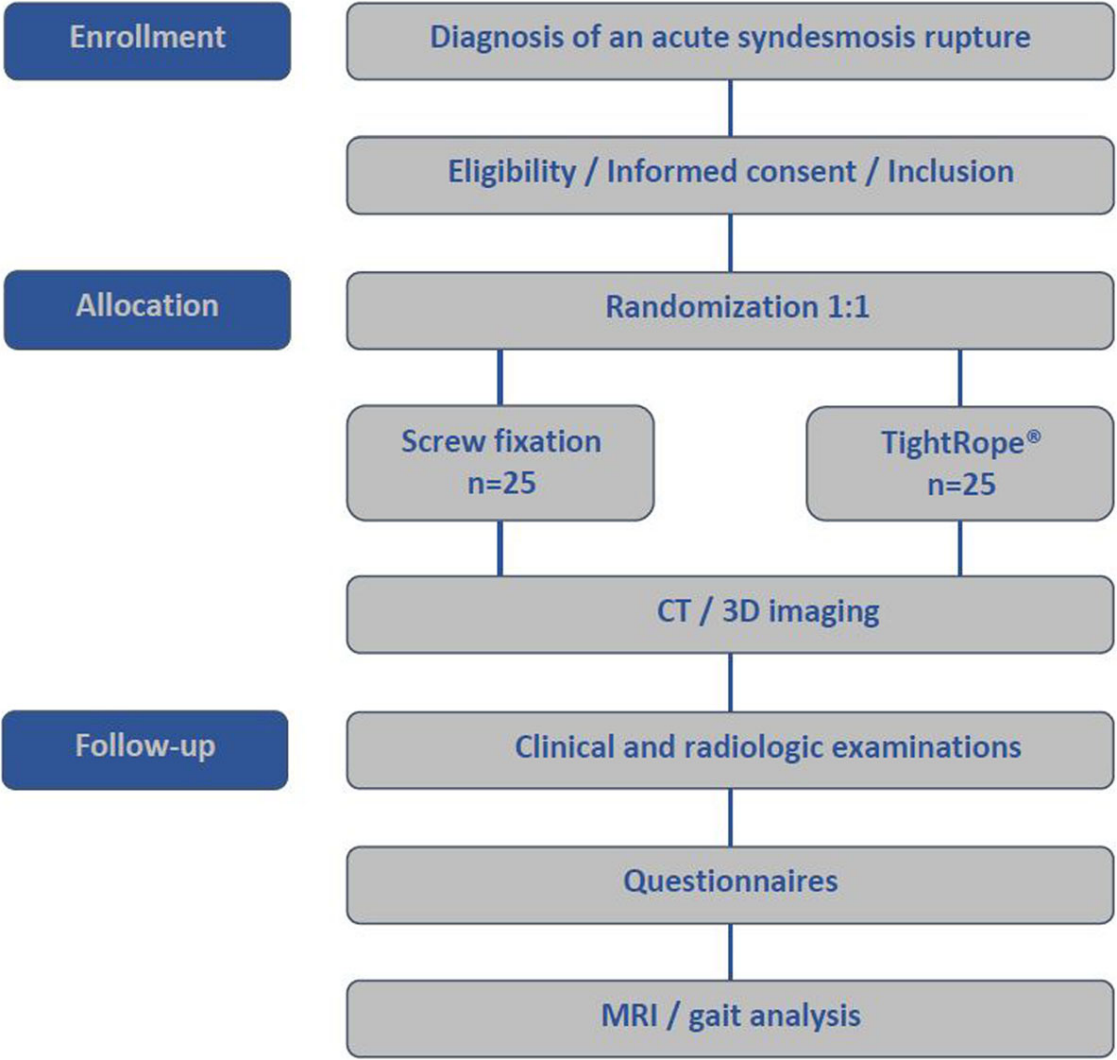
Flowchart of the study protocol

In the following, the procedure of this study is described and additionally illustrated in Fig. 1

- Diagnosis of an acute syndesmosis rupture (isolated or combined) and indication of surgical treatment in our clinic.
- Informed consent and inclusion.
- Randomization 1:1 to the different treatment options (screw fixation or TightRope®).
- Preoperative clinical and radiologic examinations.
- Preoperative clinical and psychosocial evaluations with questionnaires abovementioned.
- Surgery with screw fixation or TightRope®.
- Standardized intraoperative CT/3D imaging with ISO C 3D or CIOS Spin (Siemens Healthineers GmbH, Erlangen, Germany) for assessment of syndesmosis reduction.
- Postoperative clinical and radiologic examinations with X-ray of foot and ankle.
- Clinical and radiologic follow-up (6 weeks, 3, 6, 12 months) postoperatively + abovementioned questionnaires.
- Additional standard clinical instrumented 3D gait analysis to evaluate the biomechanical outcome (duration: 30 min) 6 months after the operation.
- Additional MRI of the ankle joint to evaluate the healing status of the syndesmosis ligament (duration 30 min) 6 months after the operation.

**Fig. 2:**
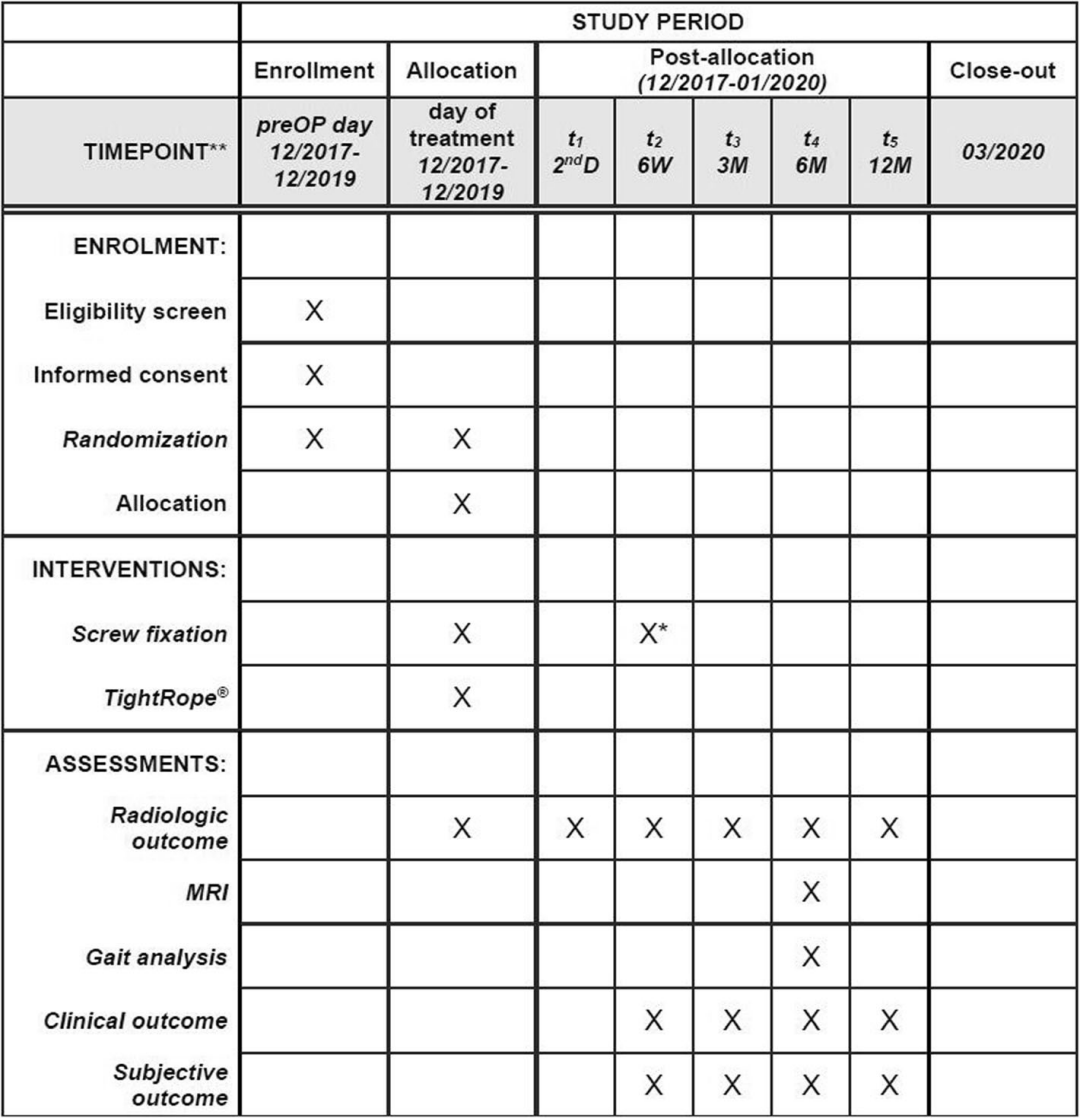
Study process schedule (according to the Standard Protocol Items: Recommendations for Interventional Trials (SPIRIT) guidelines)

### Participants and consent

All patients assigned for surgical treatment of acute syndesmosis rupture at our level 1 trauma center can be involved in our study. Patients must be at least 18 years of age without any exclusion criteria and provide their written consent before any study-relevant intervention. Before participation, each patient, suitable to the study protocol, will be fully informed by informed consent about the scientific purpose and risks associated with the procedures. The participation is voluntary and every participant is able to withdraw their consent to participate in the trial at any time without giving reasons.

Informed consent takes place in a one-to-one appointment at a trauma center with an experienced orthopedic and trauma surgeon. A full verbal explanation of the study, a written patient information sheet, and informed consent form will be provided before inclusion*.*

### Randomization

Patients will be randomly assigned to one of two groups (intervention or control) using a computer-generated random block assignment in a 1:1 ratio using nQuery Advisor v7.0 software (Statsols, Cork, Ireland). This method helps in maintaining the balance of treatment assignment while reducing the potential for selection bias. Patients, researchers performing the follow-up examination, and the trial statistician will be blinded to the group allocation. If the treatment of syndesmosis rupture is not accomplished after randomization due to an intraoperatively observed missing acute syndesmosis rupture with no need of stabilization, the patient will be excluded from final analysis. At the date of screw removal 6 to 8 weeks after the operation, a blinding of the patients will no longer be possible.

### Surgical treatment

The fractures will be fixed within 8 days of the initial trauma in both groups using standard AO (Arbeitsgemeinschaft für Osteosynthesefragen) [22] principles. Antibiotic prophylaxis will be given perioperatively. Open reduction and internal fixation (ORIF) of fibula fractures will be treated either with a 1/3 tubular plate with or without lag screws or in high fibula fractures with syndesmosis fixation only. Additional fractures of medial and/or posterior malleolus will be treated according to standard principles before stabilizing the syndesmosis. The distal tibiofibular joint will be reduced without direct visualization of the syndesmosis and held at its anatomical position by a reduction clamp. The ankle joint will be positioned at an angle of 90° between the tibial shaft and the foot during syndesmosis fixation in accordance with the randomization (cortical screw or TightRope®).

For the static syndesmotic screw fixation, a 2.5-mm hole will be drilled under fluoroscopic guidance, approximately 2 cm above and parallel to the distal tibial joint line from lateral to medial. If plating of the fibular fracture is necessary, the hole will be drilled through an empty screw hole. Three cortices will be drilled through and a 3.5-mm screw will be tightened.

For the dynamic fixation of the syndesmosis by means of TightRope®, a 3.5-mm hole will be drilled under fluoroscopic guidance, approximately 2 cm above and parallel to the distal tibial joint line (through a hole of the plate if present) from lateral to medial. A guide needle will be inserted from lateral to medial through the drill hole to position the oblong button over the medial tibial cortex and confirmed by X-ray. Afterwards, the assembly will be tensioned by pulling the free ends of the FiberWire on the lateral side. These will be hand-tied with a surgical knot and the round button will be firmly applied on the lateral cortex of the fibula (or onto the plate if present). After achieving an adequate syndesmotic fixation by either technique, the reduction clamp will be removed and the stability controlled under fluoroscopy.

After syndesmosis fixation, intraoperative CT/3D imaging with ISO C 3D or CIOS Spin (Siemens Healthineers GmbH, Deutschland) will be performed to evaluate syndesmosis reduction.

The postoperative treatment protocol is similar in both groups. The ankle will be immobilized in a below-the-knee cast with the ankle joint at 90° for 6 weeks with partial weight-bearing. Between the 6th and 8th week, only the static syndesmosis screw will be removed in a standardized outpatient surgery. The dynamic fixation of the syndesmosis by means of TightRope® needs no removal postoperatively. Afterwards, weight-bearing can be gradually increased with approximately 20 kg per week until full weight-bearing is achieved.

### Follow-up

Subsequent to surgery, all patients will receive similar follow-up procedures. Follow-up at our trauma center is standardized, based on a well-established protocol, and all procedures and diagnostics are based solely on medical indications. First radiological and clinical evaluation of the surgical treatment will be performed on day 2 after surgery. Discharge from hospital will be realized as soon as general health conditions (soft tissue conditions, patient mobility and pain level) allow it. Afterwards, patients will receive physiotherapy and manual lymphatic drainage. Further clinical and radiographic evaluations are planned for 6 weeks and 3, 6, and 12 months after surgery following our standardized procedure for fracture patients treated in our hospital (Table 1). There are no additional follow-up appointments or X-ray examinations necessary for the study. Merely the gait analysis and MRI control of the syndesmosis will be performed 6 months postoperatively during the regular follow-up appointment.

**Table 1:**
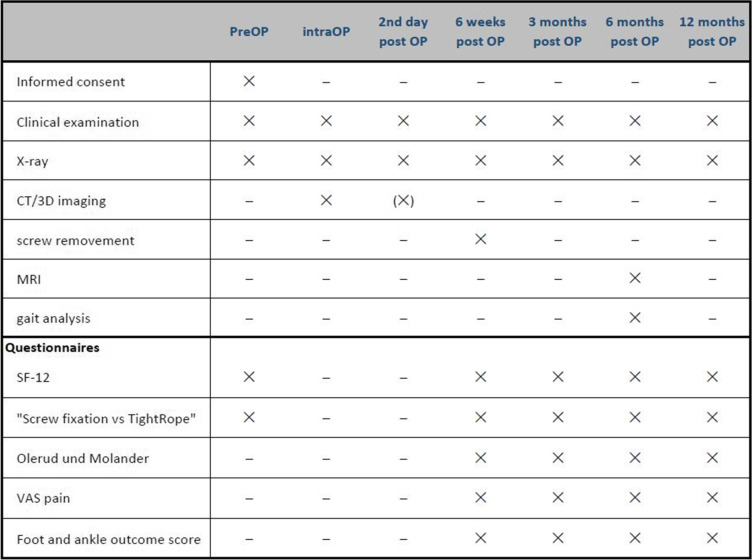
Overview of clinical and radiologic follow-up. *CT* computed tomography, *MRI* magnetic resonance imaging, *SF-12* 12-item Short Form Health Survey, *VAS pain* visual analog scale for pain

MRI images will be assessed by a senior radiologist for degenerative changes of the tibiotalar joint and the syndesmosis structure (unaffected, hyperintense signal alteration on T2, partial tear, complete tear), widening, and calcification. By using questionnaires preoperatively and postoperatively after 6 weeks and 3, 6, and 12 months during the appointments, patients can provide information on pain, mobility of foot and ankle, and quality of life (SF-12) [19] during the course of treatment. General patient data, including age, profession, body mass index (BMI), risk factors, medication, concomitant diseases, previous surgeries, and details of the accident, will be documented preoperatively.

At 25 weeks postoperatively, patients also receive a gait analysis and MRI is performed to assess motion anomalies and the healing process of the syndesmosis.

The planned study will run over 2 years. Patients will be admitted over a period of 1 year. Each patient will be treated and followed-up over a 12-month period.

All data will be stored and monitored using pseudonyms. The data of all patients will be analyzed statistically and compared at the end of the 2 years (Table 1).

Furthermore, all data of the participating patients will be carefully assessed, and all adverse events and complications of the treatment will be reported at the end of this trial. The harms will be categorized according to their degree of severity in either serious or non-serious.

### Criteria that lead to termination of study

Every participant is able to withdraw their consent to participate in the trial at any time without giving reasons. Thereby, the recorded study data may be destroyed immediately upon request or, with the consent of the participant, can still be included in the evaluation.

If initial data indicates either impossible realization due to technical difficulties or an increased risk for the participants that is potentially harmful, the study will be terminated immediately.

### Sample size calculation and statistical analyses

Formal sample size determination is not possible as potential effects are not yet known. Data from previous studies were utilized to determine the necessary sample size [23–25]. We plan a sample size of 25 patients, who will be treated with the cortical screw fixation and 25 patients, who will be treated with the dynamic TightRope® system within 2 years. The patients will be randomized to the two different treatment options. The randomization will be independent of the surgeon and the doctor who will do the examinations and the questionnaires.

The Shapiro-Wilk test will be used to check if our population is normally distributed. Standard unpaired Student’s *t* test to detect significant changes between the two groups will be conducted, if they are normally distributed. In contrast, possible differences for continuous data and scores between groups will be evaluated with the Wilcoxon *U* test, if no normal distribution exists.

The empirical distribution of continuous data and scores will be reported and calculated with means, standard deviation (SD), median, minimum, and maximum values and with absolute and relative frequencies for categoric data as well as unpaired *t*-test *p* values to depict a significant difference between the different groups. All descriptive analyses will be calculated for demographic data and for the dependent variables derived from the functional assessment, gait analyses results, and questionnaires of both groups.

An independent senior statistician of the Institute of Medical Biometry and Informatics of our university will perform the analysis. The intention-to-treat population will be used.

The analysis and illustrations will be carried out by use of SPSS version 25.0 for 135 Windows (IBM Corp., Armonk, New York, USA) and GraphPad Prism version 6.00 for Windows (GraphPad Software, San Diego, California, USA). For the representation of statistical relationships, the Pearson correlation coefficient will be used. The final details of the statistical analysis will be established in a statistical analysis plan, which will be completed before the closing of the database (before the end of data collection). A *p* value of ≤ .05 will be used to indicate statistical significance. Only a complete follow-up examination will be considered in the statistical analyses.

### Primary outcome measures

The primary endpoint of this study is a successful healing of the syndesmosis 6 months after surgery by evaluation of MRIs of the ankle [26]. MRI scanning in the plane of the syndesmotic ligaments is the investigation of choice to evaluate syndesmosis healing. MRI images will be evaluated for degenerative changes of the cartilage of the ankle, appearance (unaffected, hyperintense signal alteration on T2, partial/complete tear), widening, and calcification of the syndesmosis. If these evaluations are ordinary, successful healing can be assumed and will be classified as healed by a senior radiologist.

Another primary outcome measurement of this study is the gait analysis 6 months postoperatively to detect biomechanical differences between both treatment groups.

Due to the pilot study character, it remains to be clarified which are the most suitable parameters to depict potential effects and differences.

### Secondary outcome measures

Secondary endpoints and measurements will be recorded 6 weeks and 3, 6 and 12 months postoperatively. The measurements include different questionnaires mentioned above with subjective evaluation of the quality of life (assessed by the 12-item Short Form health survey [SF-12] questionnaire) [19] and pain (visual analog scale [VAS]). In addition, a self-designed questionnaire as well as the “Score of Olerud und Molander (OMAS)” [20] and “Foot and ankle outcome score (FAOS)” [27] will be used to evaluate the range of motion, deficits in daily life, symptoms after ankle fractures and after surgical treatment, and socioeconomic factors (period of time, returning back to work, and time of recovery).

## Discussion

The objective of this prospective randomized controlled single-center study is the comparison of the gait analysis, clinical and radiographic outcome after stabilization of an acute syndesmosis rupture with either a static implant (a 3.5-mm metallic screw through three cortices) or a dynamic device (TightRope®).

The study aims to investigate the clinical, biomechanical, and radiographic outcome of TightRope® as a dynamic stabilization device compared to the gold standard, the static screw device after acute syndesmosis ruptures of the ankle.

A lot of studies investigated the equality of both treatment options with numerous advantages of the suture button device over the screw fixation [28]. Zhang et al. described in their review that both devices had similar functional outcomes and postoperative complication rates. Furthermore, TightRope® device leads to a better range of motion and earlier return to work, compared to the screw fixation. Besides, TightRope® fixation groups had lower rates of implant removal, implant failure, and malreduction.

Actually, there is no human study with focus on objective biomechanical outcome measurements by using a gait analysis. Former biomechanical studies have demonstrated the advantages of the dynamic fixation of the syndesmosis only in animals or models [13, 29–31].

In the current study, we use the OMAS score, as it is the only validated assessment tool for ankle fractures. It detects clinical differences between treatment groups with a higher sensitivity and specificity as other scores [32].

In our study, weight-bearing is allowed after 6 weeks in both groups and only after screw removal in the intervention group, although many studies have proposed earlier weight-bearing with the dynamic fixation on the one hand [14, 33]. On the other hand, the static screw fixation prohibits early weight-bearing especially because of a high risk of screw breakage. These studies show that early weight-bearing is associated with a shorter recovery time as well as a quicker return to main activities.

Furthermore, the need and timing for postoperative routine syndesmotic screw removal are still subject to controversial discussions. In accordance with AO principles, elective routine screw removal prior to the beginning of full weight-bearing is carried out in our trauma center between the 6th and 8th week after surgery.

Unfortunately, a second operation for implant removal involves the risk of potential infections, increased costs for the patient, missed work days, a longer recovery time, or other complications [34]. The higher material costs of the TightRope® are largely surpassed by the reoperation costs [35].

Furthermore, other studies have demonstrated that early screw removal before syndesmosis healing increases the risk of developing a syndesmotic diastasis [36]. Schepers et al. demonstrated in their review that there was no better outcome when routinely removing syndesmotic screws [10].

The number of screws, screw size, and number of cortices are still subject to controversial discussions as well. According to the standard AO principles and depending on the injury, we use one or two 3.5-mm tricortical trans-syndesmotic screws as well as one or two TightRope® suture button devices.

Up to 30% of patients with an ankle fracture complain of residual symptoms such as pain, swelling, and movement restrictions. Clinical and radiological investigations cannot clearly identify the causes for these complaints. Additional objective information about the clinical outcome during postoperative follow-up examination are necessary and can be provided by modern investigation techniques such as gait analysis [37]. However, these modern investigation tools have not yet been used in the literature so far to compare the outcome of TightRope® and screw fixation patients.

Clinical gait analysis helps to identify the level of ground reaction forces, ankle loads, and reasons of incorrect loads or overloads. It is possible to determine parameters, like cadence (steps per minute), stride length, gait speed, or walking distance as well as parameters of the gait symmetry and the variability of the gait pattern. An incorrect marker placement on the body, the soft tissue’s variability, and other adjacent joint pathologies or deformities can lead to systematic measurement errors [38].

The primary strength of this study is its prospective randomized controlled and well-established design and follow-up examination protocol. All outcome measures and tools were validated. CT/3D scan will be performed to assess the quality of ankle reduction.

The large number of 50 patients is also remarkable. Moreover, only experienced orthopedic trauma surgeons will perform all surgical procedures which improve good external validity. The objective biomechanical outcome measurements with the aid of gait analysis are a unique characteristic which can show kinematics and functions of the ankle joint.

Limitations of the study include the diversity of ankle injury and fracture types that can bias the outcome. To minimize bias, all included patients will follow the same postoperative weight-bearing pattern.

We only perform CT/3D imaging on the fracture site. A bilateral CT investigation to detect possible anatomical variations will not be performed. A 1-year follow-up involves the danger of not being able to detect long-term complications as degenerative changes or osteolysis that could be a result of either a malreduced syndesmosis or an initially unreported osteochondral lesion. Therefore, a longer follow-up period would be interesting to observe such possible changes in the ankle mortise (widening, osteolysis, or osteoarthrosis).

Our hypothesis is that TightRope® will provide the same biomechanical, clinical, and radiological outcome with a similar or better postoperative function of the ankle in the gait analysis. Furthermore, we assume that the recovery time and the time required to return to work is shorter in the TightRope® cohort than in the static screw control group.

The results of the study should, in biomechanical terms, therefore help to demonstrate the comparability of different treatment methods by using the gait analysis as an objective and modern measurement method.

### Trial status

The RCT trial is ongoing (study protocol version 1.0 02.08.2017, S-454/2017), patient recruitment, and surgical treatment began in December 2017. Recruitment is expected to be completed in December 2019. The follow-up is conducted over a 12-month period for each patient included. Data analysis will only be performed after complete 1-year follow-up. Thereafter, the final results will be published.

### Study beginning

The acquisition of study participants started after receiving the approval of the trial protocol by the local ethics committee.

## Supplementary information

**Additional file 1.** Standard Protocol Items: Recommendations for Interventional Trials (SPIRIT) 2013 Checklist: recommended items to address in a clinical trial protocol and related documents.

## Data Availability

The datasets used and analyzed during the current study are available from the corresponding author on reasonable request.

## Abbreviations

AO: “Arbeitsgemeinschaft für Osteosynthesefragen”
BMI: Body mass index
CT: Computed tomography
MRI: Magnetic resonance imaging
OMAS: Score of Olerud und Molander
ORIF: Open reduction and internal fixation
SF-12: 12-item Short Form Health Survey
VAS pain: Visual analog scale for pain

## References

[1] Weening B, Bhandari M (2005). Predictors of functional outcome following transsyndesmotic screw fixation of ankle fractures. J Orthop Trauma.

[2] Lindsjo U (1981). Operative treatment of ankle fractures. Acta Orthop Scand Suppl.

[3] Laflamme M, Belzile EL, Bedard L, van den Bekerom MP, Glazebrook M, Pelet S (2015). A prospective randomized multicenter trial comparing clinical outcomes of patients treated surgically with a static or dynamic implant for acute ankle syndesmosis rupture. J Orthop Trauma.

[4] Brown KW, Morrison WB, Schweitzer ME, Parellada JA, Nothnagel H (2004). MRI findings associated with distal tibiofibular syndesmosis injury. AJR Am J Roentgenol.

[5] Valderrabano V, Leumann A, Pagenstert G, Frigg A, Ebneter L, Hintermann B (2006). Chronic ankle instability in sports -- a review for sports physicians. Sportverletz Sportschaden.

[6] Leeds HC, Ehrlich MG (1984). Instability of the distal tibiofibular syndesmosis after bimalleolar and trimalleolar ankle fractures. J Bone Joint Surg Am.

[7] Rammelt S, Zwipp H, Grass R (2008). Injuries to the distal tibiofibular syndesmosis: an evidence-based approach to acute and chronic lesions. Foot Ankle Clin.

[8] Sagi HC, Shah AR, Sanders RW (2012). The functional consequence of syndesmotic joint malreduction at a minimum 2-year follow-up. J Orthop Trauma.

[9] Gardner MJ, Demetrakopoulos D, Briggs SM, Helfet DL, Lorich DG (2006). Malreduction of the tibiofibular syndesmosis in ankle fractures. Foot Ankle Int..

[10] Schepers T (2011). To retain or remove the syndesmotic screw: a review of literature. Arch Orthop Trauma Surg.

[11] Bava E, Charlton T, Thordarson D (2010). Ankle fracture syndesmosis fixation and management: the current practice of orthopedic surgeons. Am J Orthop (Belle Mead NJ).

[12] Schepers T (2012). Acute distal tibiofibular syndesmosis injury: a systematic review of suture-button versus syndesmotic screw repair. Int Orthop.

[13] Soin SP, Knight TA, Dinah AF, Mears SC, Swierstra BA, Belkoff SM (2009). Suture-button versus screw fixation in a syndesmosis rupture model: a biomechanical comparison. Foot Ankle Int..

[14] Thornes B, Shannon F, Guiney AM, Hession P, Masterson E (2005). Suture-button syndesmosis fixation: accelerated rehabilitation and improved outcomes. Clin Orthop Relat Res.

[15] Sanders D, Schneider P, Taylor M, Tieszer C, Lawendy AR (2019). Canadian Orthopaedic Trauma S. Improved reduction of the tibiofibular syndesmosis with TightRope compared with screw fixation: results of a randomized controlled study. J Orthop Trauma.

[16] McKenzie AC, Hesselholt KE, Larsen MS, Schmal H (2019). A systematic review and meta-analysis on treatment of ankle fractures with Syndesmotic rupture: suture-button fixation versus cortical screw fixation. J Foot Ankle Surg..

[17] Oppelt K, Hogan A, Stief F, Grutzner PA, Trinler U. Movement analysis in orthopedics and trauma surgery - measurement systems and clinical applications. Z Orthop Unfall. 2020;158(3):304-17. 10.1055/a-0873-1557 31291674

[18] Baker R, Esquenazi A, Benedetti MG, Desloovere K (2016). Gait analysis: clinical facts. Eur J Phys Rehabil Med.

[19] Busija L, Pausenberger E, Haines TP, Haymes S, Buchbinder R, Osborne RH (2011). Adult measures of general health and health-related quality of life: Medical Outcomes Study Short Form 36-Item (SF-36) and Short Form 12-Item (SF-12) Health Surveys, Nottingham Health Profile (NHP), Sickness Impact Profile (SIP), Medical Outcomes Study Short Form 6D (SF-6D), Health Utilities Index Mark 3 (HUI3), Quality of Well-Being Scale (QWB), and Assessment of Quality of Life (AQoL). Arthritis Care Res (Hoboken).

[20] Olerud C, Molander H (1984). A scoring scale for symptom evaluation after ankle fracture. Arch Orthop Trauma Surg.

[21] Roos EM, Roos HP, Ekdahl C, Lohmander LS (1998). Knee injury and osteoarthritis outcome score (KOOS)--validation of a Swedish version. Scand J Med Sci Sports.

[22] Hodgson S (2009). AO principles of fracture management. Ann R Coll Surg Engl.

[23] Andersen MR, Frihagen F, Hellund JC, Madsen JE, Figved W (2018). Randomized trial comparing suture button with single syndesmotic screw for syndesmosis injury. J Bone Joint Surg Am.

[24] Cottom JM, Hyer CF, Philbin TM, Berlet GC (2009). Transosseous fixation of the distal tibiofibular syndesmosis: comparison of an interosseous suture and endobutton to traditional screw fixation in 50 cases. J Foot Ankle Surg.

[25] Kortekangas T, Savola O, Flinkkila T, Lepojarvi S, Nortunen S, Ohtonen P (2015). A prospective randomised study comparing TightRope and syndesmotic screw fixation for accuracy and maintenance of syndesmotic reduction assessed with bilateral computed tomography. Injury..

[26] Kellett JJ, Lovell GA, Eriksen DA, Sampson MJ (2018). Diagnostic imaging of ankle syndesmosis injuries: a general review. J Med Imaging Radiat Oncol.

[27] Roos EM, Brandsson S, Karlsson J (2001). Validation of the foot and ankle outcome score for ankle ligament reconstruction. Foot Ankle Int..

[28] Zhang P, Liang Y, He J, Fang Y, Chen P, Wang J (2017). A systematic review of suture-button versus syndesmotic screw in the treatment of distal tibiofibular syndesmosis injury. BMC Musculoskelet Disord.

[29] Miller RS, Weinhold PS, Dahners LE (1999). Comparison of tricortical screw fixation versus a modified suture construct for fixation of ankle syndesmosis injury: a biomechanical study. J Orthop Trauma.

[30] Thornes B, Walsh A, Hislop M, Murray P, O'Brien M (2003). Suture-endobutton fixation of ankle tibio-fibular diastasis: a cadaver study. Foot Ankle Int.

[31] Klitzman R, Zhao H, Zhang LQ, Strohmeyer G, Vora A (2010). Suture-button versus screw fixation of the syndesmosis: a biomechanical analysis. Foot Ankle Int..

[32] Button G, Pinney S (2004). A meta-analysis of outcome rating scales in foot and ankle surgery: is there a valid, reliable, and responsive system?. Foot Ankle Int..

[33] Degroot H, Al-Omari AA, El Ghazaly SA (2011). Outcomes of suture button repair of the distal tibiofibular syndesmosis. Foot Ankle Int..

[34] Andersen MR, Frihagen F, Madsen JE, Figved W (2015). High complication rate after syndesmotic screw removal. Injury..

[35] Lalli TA, Matthews LJ, Hanselman AE, Hubbard DF, Bramer MA, Santrock RD (2015). Economic impact of syndesmosis hardware removal. Foot (Edinb).

[36] Juarez-Jimenez HG, Garibay-Cervantes A, Rosas-Medina JA, Salas-Morales GA, Rodriguez-Reyes EJ (2018). Prevalence of complications related to the removal of the syndesmotic screw. Acta Ortop Mex.

[37] Wright CJ, Arnold BL, Coffey TG, Pidcoe PE (2011). Repeatability of the modified Oxford foot model during gait in healthy adults. Gait Posture.

[38] Mittlmeier T, Rosenbaum D (2005). Clinical gait analysis. Unfallchirurg..

[39] Chan AW, Tetzlaff JM, Gotzsche PC, Altman DG, Mann H, Berlin JA (2013). SPIRIT 2013 explanation and elaboration: guidance for protocols of clinical trials. BMJ..

[40] Schulz KF, Altman DG, Moher D, Group C (2011). CONSORT 2010 statement: updated guidelines for reporting parallel group randomised trials. Int J Surg.

